# Harnessing Machine Learning to Predict Methadone Overdose Risk: Insights from Illinois's SUDORS Data

**DOI:** 10.23889/ijpds.v9i5.2554

**Published:** 2024-09-18

**Authors:** Yingxuan Liu, Maryann Mason

**Affiliations:** 1 Northwestern University Feinberg School of Medicine

Knowledge of decedent characteristics associated with methadone involvement in unintentional drug overdose deaths can help to inform efforts to intervene in these deaths.  ML models with existing data, offer a cost-effective means to help create effective interventions. In this study, we aim to develop an optimized ML algorithm for predicting overdose death patterns due to methadone using data from Illinois's State Unintentional Drug Overdose Reporting System (SUDORS).

We utilized IL SUDORS 2019-2022 data for training (n = 11931) with selected indicators (n = 28). To address the imbalance in non-methadone-related deaths, we applied the Synthetic Minority Oversampling Technique. Next, we assessed various machine learning models, including logistic regression, support vector machine, random forest, neural network, and ridge regression. Model performance was evaluated using metrics such as accuracy, precision, area under the precision-recall curve (AUPRC), etc.

Utilizing separate training (n = 15813), validation (n = 3388), and test (n = 3389) datasets, the Random Forest Model outperforms all other models with 92% accuracy, 91% precision, 93% recall, and 0.97 AUPRC. Notably, these outcomes were achieved using primarily demographic indicators. Through analyzing partial dependence plots, we were able to see how changes in each indicator dynamically influence methadone-related fatalities.

This study demonstrates the potential of ML models in identification of methadone involvement in unintentional drug overdose deaths and contributes to the knowledge base for the prevention of these deaths. Leveraging SUDORS data, the Random Forest Model exhibited exceptional performance, highlighting its value for healthcare professionals, prevention and harm reduction specialists, and policymakers.

